# MSFN-YOLOv11: A Novel Multi-Scale Feature Fusion Recognition Model Based on Improved YOLOv11 for Real-Time Monitoring of Birds in Wetland Ecosystems

**DOI:** 10.3390/ani15233472

**Published:** 2025-12-02

**Authors:** Linqi Wang, Lin Ye, Xinbao Chen, Nan Chu

**Affiliations:** 1 School of Earth Sciences and Spatial Information Engineering, Hunan University of Sciences and Technology, Xiangtan 411201, China; 2 Institute of Tibetan Plateau Research, Chinese Academy of Sciences, Beijing 100101, China

**Keywords:** deep learning, bird recognition, MSFN-YOLOv11, multi-scale feed-forward neural network (MSFN), object detection

## Abstract

Intelligent bird species recognition is vital for biodiversity monitoring and ecological conservation. This study tackles the challenge of declining recognition accuracy caused by occlusions and imaging noise in complex natural environments. Focusing on ten representative bird species from the Dongting Lake Wetland, we propose an improved YOLOv11n-based model named MSFN-YOLO11, which incorporates multi-scale feature fusion. After selecting YOLOv11n as the baseline through comparison with the most-stable version of YOLOv8n, we enhance its backbone by introducing an MSFN module. This module strengthens global and local feature extraction via parallel dilated convolution and a channel attention mechanism. Experiments are conducted on a self-built dataset containing 4540 images of ten species with 6824 samples. To simulate real-world conditions, 25% of samples are augmented using random occlusion, Gaussian noise (σ = 0.2, 0.3, 0.4), and Poisson noise. The improved model achieves a *mAP*@50 of 96.4% and *mAP*@50-95 of 83.2% on the test set. Although the *mAP*@50 shows a slight improvement of 0.3% compared to the original YOLOv11, it has contributed to an 18% reduction in training time. Furthermore, it also demonstrates practical efficacy in processing dynamic video, attaining an average 63.1% accuracy at 1920 × 1080@72fps on an NVIDIA_Tesla_V100_SXM2_32_GB. The proposed model provides robust technical support for real-time bird monitoring in wetlands and enhances conservation efforts for endangered species.

## Simple Summary

This study addresses the challenge of accurately identifying bird species in complex environments where occlusion and noise diminish recognition accuracy. We developed an enhanced YOLOv11 model incorporating a Multi-Scale Feed-forward Network (MSFN) to improve robustness. The model was trained on a dedicated dataset comprising 4540 images with 6824 samples in wetland ecosystems and evaluated under synthetic noise conditions, achieving a *mAP*@50 of 96.4% and demonstrating improved recall in noisy situations. Furthermore, it facilitated effective species identification in dynamic video sequences, showcasing significant potential for supporting real-time biodiversity monitoring and the conservation of endangered bird species.

## 1. Introduction

Birds are pivotal indicator species for assessing ecosystem health and biodiversity. Their population dynamics offer critical insights into the status of wetland ecosystems [1]. Taking Dongting Lake, a critical node on the East Asian-Australasian flyway, as an example, it provides habitats for numerous rare species such as the Oriental Stork and the White-tailed Eagle [2,3]. However, conducting effective bird monitoring in these areas is extremely challenging due to the complex wetland environment, which is characterized by cluttered backgrounds (e.g., water surfaces and reed beds), frequent occlusions, variable lighting, and the inherent flocking behavior of birds. These challenges severely limit the accuracy and efficiency of both traditional manual surveys and conventional automated methods, creating a pressing demand for robust, automated recognition technology to advance intelligent ecological conservation.

Traditional bird monitoring methods, such as manual field observation and infrared camera trapping, are hampered by low efficiency, limited coverage, and high susceptibility to environmental conditions [2]. Currently, the advent of deep learning using computer vision has catalyzed a paradigm shift in bird detection, transitioning from traditional manual feature-based methods to deep neural networks [4,5,6,7]. These deep learning-based detectors are broadly categorized into two-stage and single-stage models [8]. While two-stage detectors (e.g., Faster R-CNN [9], Mask R-CNN [10], Light weight CNN [11]) achieve high accuracy, their substantial computational cost hinders real-time deployment. In contrast, mainstream single-stage models like the YOLO series strike an optimal balance between speed and accuracy, making them suitable for real-time applications [8]. Consequently, YOLO series models have been widely adopted and tailored for bird monitoring in various scenarios [12,13,14,15].

The evolution of the YOLO series is marked by continuous architectural optimization [16] and, more importantly, by scenario-specific adaptations [17]. For complex environments like wetlands, enhancements primarily target multi-scale feature fusion and noise robustness. For instance, to handle varying bird sizes and cluttered backgrounds, Ma et al. [18] integrated a Receptive-Field Attention convolution and a dynamic feature fusion network into YOLOv8 for Poyang Lake. Similarly, Yang et al. [19] developed YOLO-AWK based on YOLOv11n, introducing an in-scale feature interaction module for farmland settings. These efforts align with broader technical trends, such as employing attention mechanisms (e.g., BiFormer [3]) and designing novel feature fusion modules (e.g., SSFF [15]) to boost performance on small objects.

A critical, yet under-explored, challenge lies in the robustness of these models against the composite disturbances inherent in dynamic wetland video streams [6]. While some studies have addressed noise through data augmentation (e.g., simulating motion blur) or video frame processing (e.g., panorama stitching) [6,11,20,21], these strategies often target relatively singular or specific noise types. In reality, practical surveillance involves the coexistence and interaction of multiple degradation factors—such as motion blur, water surface glare, and dense occlusion. Most existing enhanced models [12,13,14,15,18,19] are validated primarily on static images, leaving a significant robustness gap for deployment in real-world, dynamic scenarios [22,23]. Furthermore, there is a lack of a systematic framework that can construct a unified and robust feature representation capable of simultaneously handling multi-scale targets, complex backgrounds, and composite imaging noise.

To bridge these gaps, this study proposes MSFN-YOLOv11, a novel multi-scale feature fusion model based on an improved YOLOv11, specifically designed for the challenging conditions of wetland bird monitoring. The main contributions of this work are fourfold: (1) We propose a novel Multi-Scale Feed-forward Network (MSFN) module that leverages parallel dilated convolutions and channel attention to enhance multi-scale contextual feature representation and discriminative capability in complex scenes. (2) We construct a high-quality, region-specific dataset for wetland bird monitoring and employ a targeted mixed-noise augmentation strategy to better simulate real-world composite disturbances. (3) Through comprehensive experiments, we demonstrate that our model achieves superior performance in recognition accuracy and robustness, particularly in noisy and occluded scenarios, while also improving training efficiency. (4) We validate the model’s effectiveness on real surveillance videos, confirming its potential as a reliable technical solution for practical intelligent bird monitoring systems in wetlands.

## 2. Materials and Methods

### 2.1. Overview of the Methodology

**Figure 1:**
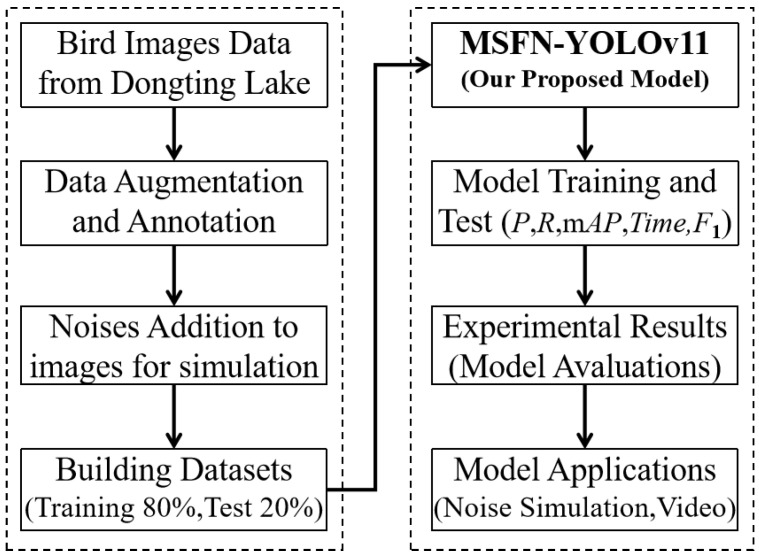
The overall flowchart of methodology.

The overall technical workflow of the proposed methods is illustrated in Figure 1. This study focuses on ten bird species from Dongting Lake, utilizing images collected from online repositories and field investigations conducted with drones. All images were annotated using LabelImg. During the data preprocessing stage, duplicate images were removed, and data augmentation techniques were applied to enhance the diversity of the dataset. Furthermore, to simulate real-world image degradation, Gaussian noise (with σ values of 0.2, 0.3, and 0.4) and Poisson noise were added to 25% of the images. The cleaned dataset was then divided into training and testing subsets at an 80:20 ratio, resulting in 5402 training samples and 1422 test samples. For bird species identification, YOLO-based object detection models were adopted. Specifically, the YOLOv8 and YOLOv11 architectures were trained and evaluated. The superior model—YOLOv11—was further improved by integrating an MSFN to aggregate multi-scale features and enhance nonlinear representation. The performance of the proposed MSFN-YOLOv11 model was rigorously evaluated using metrics such as precision (*P*), recall (*R*), mean Average Precision (*mAP*), inference time, and *F*1-score. Experimental results, including noise simulation and video-based applications, demonstrate the effectiveness of the proposed approach.

### 2.2. Birds Dataset

**Figure 2:**
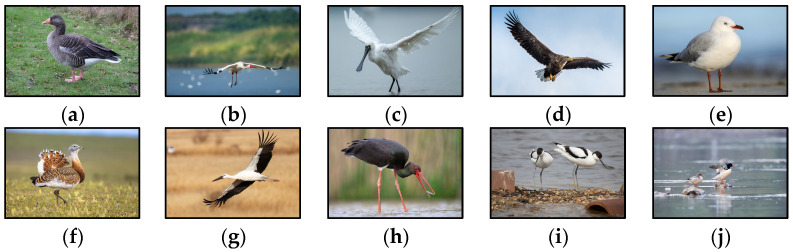
Examples of typical bird species in our collected dataset. (**a**) Greater White-fronted Goose; (**b**) White Crane; (**c**) Black-faced Spoonbill; (**d**) White-tailed Sea Eagle; (**e**) Common Black-headed Gull; (**f**) Great Bustard; (**g**) Oriental Stork; (**h**) Black Stork; (**i**) Pied Avocet; (**j**) Scaly-sided Merganser.

The performance of bird species identification is highly contingent upon the scale, quality, and diversity of the training dataset. Birds exhibit highly variable behaviors—such as diverse flight patterns and perching poses—and are significantly influenced by environmental factors including lighting, complex backgrounds, and occlusion. These variations often lead to substantial performance degradation when models encounter unseen scenarios. Therefore, it is imperative to develop a dataset that incorporates a wide range of species, behavioral states, and environmental contexts to enhance both the generalization capacity and recognition accuracy of the model. Widely used public datasets like the Caltech Birds [24] and HiFSOD-Bird [25], while valuable for general bird recognition, are not designed to capture the specific ecological context of the Dongting Lake region. Their limited relevance to local species diminishes their utility for training models aimed at regional conservation. To address this research gap and facilitate in-depth studies of regional avifauna, this work presents a purpose-built, custom-curated dataset comprising 4540 images with 6824 annotated samples across ten characteristic bird species most commonly found in Dongting Lake. The data were sourced from multiple platforms, including the China Bird Watch Center (www.birdreport.cn/, accessed on the 1 April 2025), Droit Vision (www.droitstock.com, accessed on the 5 April 2025), and the China Wild Bird Photo Gallery (www.szbird.org.cn/new/0_cnbird/first_pc.html, accessed on the 5 April 2025). The dataset contains natural scenes of different bird species, the same species from various angles and forms, and under different lighting conditions, as illustrated in Figure 2.

This study focused on ten typical bird species commonly found in Dongting Lake: the black stork (B1), black-faced spoonbill (B2), red-billed gull (B3), great bustard (B4), greater white-fronted goose (B5), oriental white stork (B6), pied avocet (B7), Chinese merganser (B8), White Crane (B9), and white-tailed eagle (B10). Bird instances were manually annotated using LabelImg software(v1.8.5) to create a custom object detection dataset. The labeling process adhered to a unified standard, which included both species attributes and bounding box localization. To ensure effective data utilization, the dataset was partitioned into training and test sets in an 8:2 ratio. The corresponding image counts for the training and testing samples are presented in Table 1.

### 2.3. Data Augmentation

In real-world scenarios, bird images are often subjected to various forms of interference, including sensor inaccuracies, variations in illumination, and transmission disturbances, all of which contribute to image degradation. For instance, during rainy or foggy weather, atmospheric particles such as water droplets and aerosols cause Mie scattering, which reduces image contrast. Raindrops may manifest as linear noise streaks ranging from 5 to 15 pixels in length, while impacts on the lens can introduce motion blur. Additionally, image quality is severely degraded by significant noise when shooting with a high ISO in low-light conditions at dawn or dusk [24]. Notably, each doubling of the ISO value approximately doubles the standard deviation of Gaussian noise [25]. To simulate real-world degradations and enhance recognition performance under conditions of blur or low contrast, as well as to improve algorithmic robustness across diverse environments, this study introduced varying levels of Gaussian noise combined with Poisson noise to 25% of the dataset, which consists of a total of 1065 images with 1706 samples. This augmentation emulates typical natural-scene interference, thereby reducing the risk of overfitting to idealized noise-free data. By simulating these degradations, the algorithm can proactively adapt to complex interference patterns while preserving critical avian recognition features such as feather patterns, texture, and silhouette.

Gaussian noise follows a normal distribution, with its intensity governed by the standard deviation (σ) and mean (μ) parameters, as illustrated in Equation (1). The mean (μ) is set to 0, indicating that the pixel values are not offset. A larger standard deviation (σ) results in greater noise intensity.

(1) $$f(x)=\frac{1}{\sigma \sqrt{2\pi }}e(-\frac{{\left(x-\mu \right)}^{2}}{2\sigma^{2}})$$

In this study, three intensities of Gaussian noise were introduced (see Table 2). Mild Gaussian noise simulates slight low-light conditions, such as those experienced during twilight and dawn. Medium Gaussian noise represents typical low-light environments, including overcast days and shaded areas beneath trees. Conversely, heavy Gaussian noise simulates extreme low-light scenarios, such as imaging with infrared fill light at night.

**Figure 3:**
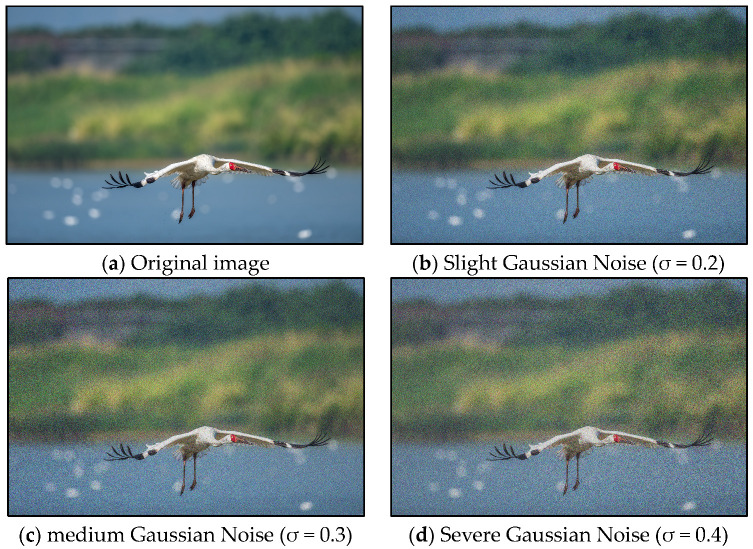
Gaussian Noise sample (this is a sample of White Crane).

Here, an example of Gaussian noise augmentation is illustrated in Figure 3. Gaussian noise is particularly relevant in outdoor bird imagery captured by drones or telephoto lenses, as it manifests as fine-grained, snow-like artifacts that uniformly affect the texture of the images. Importantly, it does not obscure key ornithological features and effectively simulates sensor noise in low-light conditions.

Meanwhile, Poisson noise, commonly referred to as shot noise, is characterized by statistically indeterminate fluctuations in the photon counting process. In low-light conditions, both the arrival times and the number of photons are random, resulting in noise on the image sensor. For example, in photography, using a high ISO setting or shooting at night leads to insufficient light, which reduces the number of photons captured by the sensor and consequently decreases the signal-to-noise ratio. As a result, Poisson noise becomes more pronounced under these conditions. For further details, please refer to Formula (2).

(2) $$P\left(x=k\right)=\frac{e^{-\lambda }\lambda^{k}}{k!}$$

**Figure 4:**
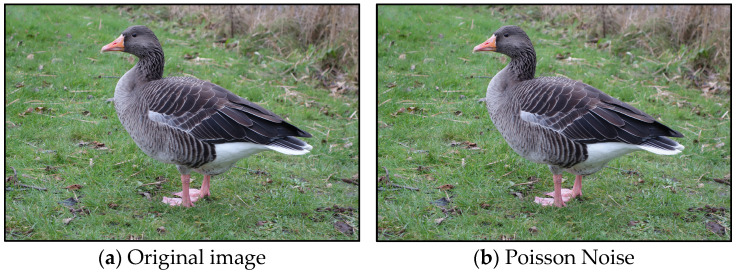
Poisson noise Sample (this is a sample of Greater White-fronted Goose).

The probability distribution of Poisson noise follows a Poisson distribution, where the variance is equal to the mean. Therefore, in low-light conditions, the variance of the noise increases with the signal intensity; however, the signal-to-noise ratio improves with the square root of the signal intensity. This means that as the signal strength increases, the prominence of the noise also becomes more pronounced. The intensity of the pixel values fundamentally influences the noise. An example of Poisson noise is illustrated in Figure 4.

### 2.4. The Original YOLOv11 Network

**Figure 5:**
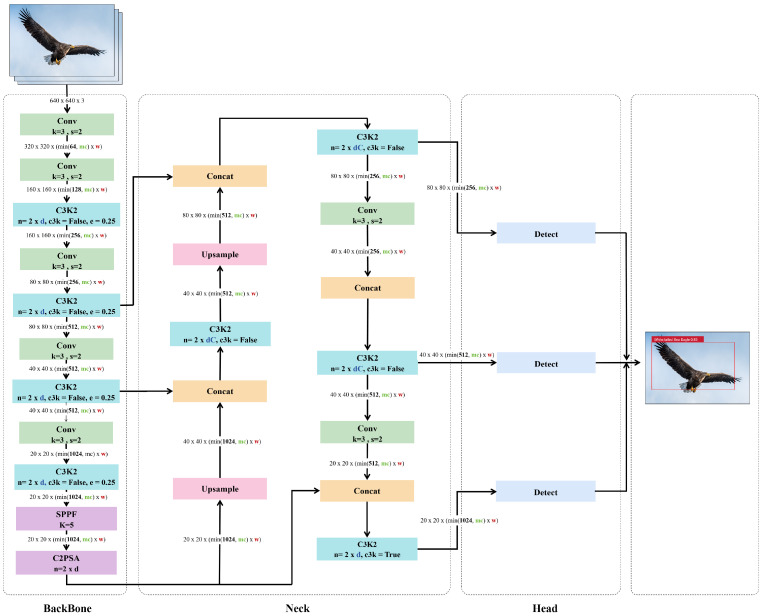
YOLOv11 network structure [16]. (1) The YOLOv11 model is composed of three primary components: the backbone, the neck, and the head. (2) Key modules include C3k2, SPPF, C2PSA, and a decoupled detection head. The C3k2 module performs efficient feature extraction, SPPF integrates multi-scale contextual information, C2PSA enhances spatial attention distribution, and the head performs classification and regression tasks. (3) The concatenation module (Concat) is employed to fuse feature maps from different scales along a specified dimension.

YOLOv11 demonstrates a superior speed–accuracy trade-off, particularly in real-time monitoring under complex environmental conditions. The model architecture consists of three core components: a backbone network, a feature fusion module, and a detection head (Figure 5). In the backbone, YOLOv11 introduces a novel C3K2 module (Cross-Stage Partial with Kernel Size 2), which reduces computational costs by employing smaller convolutional kernels while maintaining representational capacity compared to the C2f structure in YOLOv8. The model retains the SPPF (Spatial Pyramid Pooling–Fast) module to integrate local and global context through multi-scale pooling, thereby enhancing perception across varying object sizes.

During feature fusion, YOLOv11 incorporates a C2PSA (Convolutional Block with Parallel Spatial Attention) module, which introduces a spatial attention mechanism to enhance feature extraction, particularly for small and occluded targets in cluttered scenes, while maintaining high inference efficiency. The detection head employs a decoupled structure that optimizes classification, regression, and confidence tasks separately, thereby reducing interference among sub-tasks and improving overall accuracy. Furthermore, YOLOv11 adopts an anchor-free approach [26], where each grid cell directly predicts the bounding box coordinates $\left(b_{x},b_{y},b_{w},b_{h}\right)$ and the objectness confidence without predefined anchor boxes. The coordinate prediction is carried out as follows:

(3) $$b_{x}=\sigma (t_{x})+c_{x},\;b_{y}=\sigma (t_{y})+c_{y},\;b_{w}=e^{t_{w}}\cdot p_{w},\;b_{h}=e^{t_{h}}\cdot p_{h}$$

The objectness and class probabilities are obtained as follows:

(4) $$P_{\mathrm{obj}}=\sigma (t_{o}),\;P_{\mathrm{cls}}=\mathrm{softmax}(t_{c})$$

To achieve a more flexible, task-adaptive optimization, the overall training objective combines the bounding box regression loss, confidence loss, and classification loss:

(5) $$L_{\mathrm{total}}=\lambda_{\mathrm{box}}\cdot L_{\mathrm{box}}+\lambda_{\mathrm{conf}}\cdot L_{\mathrm{conf}}+\lambda_{\mathrm{cls}}\cdot L_{\mathrm{cls}}$$

where the bounding box loss is defined by the Complete IoU (CIoU) metric to ensure geometric consistency:

(6) $$L_{\mathrm{box}}=1-\mathrm{IoU}+\frac{\rho^{2}(b,b_{gt})}{c^{2}}+\alpha \cdot v$$

This anchor-free design and CIoU-based optimization improve convergence speed and robustness in dense or cluttered scenes. The model inherently supports multi-task learning, including object detection, instance segmentation, keypoint detection, and rotated bounding box detection [27], thus providing strong extensibility for multi-modal ecological monitoring applications.

### 2.5. The Enhanced YOLOv11 Network

**Figure 6:**
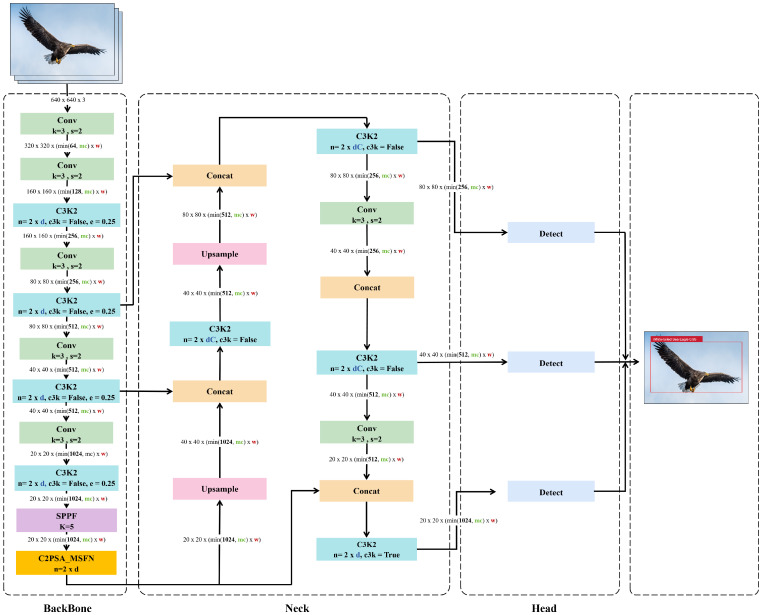
The Enhanced YOLOv11 network structure, modified from [16]. It replaces the FFN layer in the original C2PSA module with MSFN, forming a new C2PSA_MSFN module.

In this study, we propose an enhanced algorithm based on YOLOv11 for recognizing rare bird species in Dongting Lake. While maintaining the overall hierarchical feature processing framework of YOLOv11, we introduce structural optimizations into the core modules of the backbone network. Specifically, the Feed-forward Neural Network (FFN) layer in the original C2PSA module is replaced with a MSFN, resulting in a new composite module termed C2PSA_MSFN. This enhancement significantly improves the model’s ability to process global and local features across multiple scales. Following optimization, the C2PSA_MSFN module facilitates hierarchical multi-scale feature extraction and cross-layer integration while preserving computational efficiency. It effectively addresses the limited representational capacity of the original FFN layer, thereby providing more discriminative features for subsequent fusion in the neck network and enhancing object recognition in the detection head. Collectively, these improvements elevate the model’s detection performance in complex ecological environments. The enhanced YOLOv11, namely MSFN-YOLO11, is illustrated in Figure 6.

**Figure 7:**
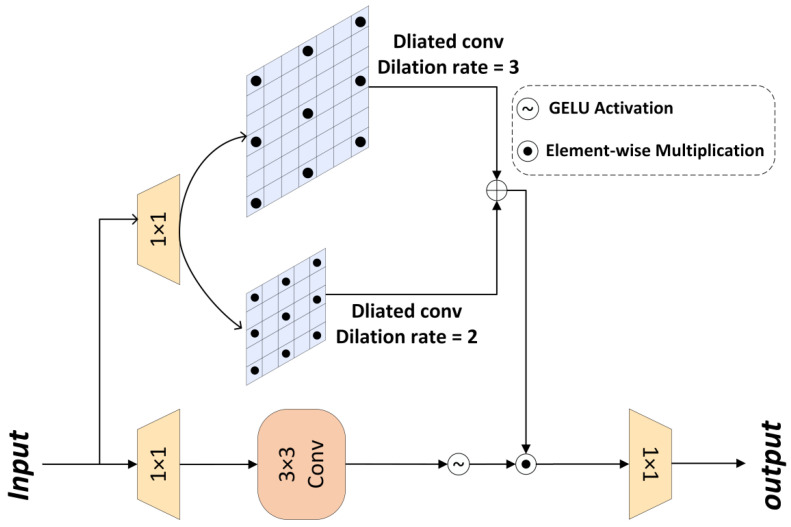
MSFN Architecture [28].

The details of the MSFN are illustrated in Figure 7. It initially expands the feature channels through two 1 × 1 convolutions with an expansion ratio of *γ* = 2. The input features are then processed through two parallel paths: the lower branch employs depthwise convolution for local feature extraction, while the upper branch utilizes multi-scale dilated convolution to capture broader contextual information. A gating mechanism integrates both paths through element-wise multiplication, thereby enhancing the capacity for nonlinear transformation.

For the input tensor $X\in R^{\hat{H}\times \hat{W}\times \hat{C}}$, the definition of MSFN is given in Equations (7) and (8).

(7) $$Gating\left(X\right)=\phi \left(W_{3\times 3\times 3}W_{1\times 1}\left(X\right)\right)⨀W_{3\times 3}^{2}W_{1\times 1}\left(X\right)+W_{3\times 3}^{3}W_{1\times 1}\left(X\right)\;$$

(8) $$X_{out}=W_{1\times 1}Gating\left(X\right)\;$$

Here, ⨀  denotes element-wise multiplication, ϕ represents the GELU nonlinear activation function, $W_{3\times 3}^{2}\;$ denotes a 3 × 3 dilated convolution with a dilation rate of 2, and $W_{3\times 3}^{3}\;$ denotes a 3 × 3 dilated convolution with a dilation rate of 3.

### 2.6. Assessment Metrics

To comprehensively evaluate the model’s performance, a set of standard metrics for object detection was employed, including Precision (*P*), Recall (*R*), mean Average Precision(*mAP*) at IoU = 0.5 (*mAP*@50), mean Average Precision over IoU thresholds from 0.5 to 0.95 (*mAP*@50:95), and the *F*1 score. The fundamental concepts are defined as follows:

- True Positive (*TP*): The number of positive instances correctly detected by the model.
- False Positive (*FP*): The number of negative instances incorrectly detected as positive.
- False Negative (*FN*): The number of positive instances that the model failed to detect.
- True Negative (*TN*): The number of negative instances correctly detected as negative.

Based on these definitions, the primary metrics are calculated as follows [29]:

Accuracy (*A*) is to measure the total percentage of correct classifications (Equation (9)).

(9) $$A=\frac{TP+TN}{TP+TN+FN+FP}$$

Precision (*P*) measures the reliability of the model’s positive predictions, representing the proportion of correct detections among all predicted positives (Equation (10)).

(10) $$P=\frac{TP}{TP+FP}$$

Recall (*R*) measures the model’s ability to identify all relevant targets, representing the proportion of actual positives that are correctly detected (Equation (11)).

(11) $$R=\frac{TP}{TP+FN}$$

Intersection over Union (*IoU*) quantifies the spatial overlap between a predicted bounding box and its corresponding ground-truth box (Equation (12)). It is the basis for determining a correct detection.

(12) $$IoU=\frac{Area\;of\;Overlap}{Area\;of\;Union}$$

The mean Average Precision (*mAP*) is the primary metric for object detection. Its calculation is carried out as follows:

- The Average Precision (*AP*) is computed for each object class based on the Precision-Recall curve.
- *mAP@50* is the mean of AP across all classes at a single IoU threshold of 0.5. It is suitable for scenarios with moderate localization requirements.
- *mAP@50:95* is a more stringent metric, defined as the average *mAP* computed at multiple IoU thresholds from 0.5 to 0.95 in steps of 0.05. It imposes a higher demand on bounding box accuracy.

Given the need for precise localization in ecological monitoring, this study adopts *mAP*@50:95 as the primary benchmark for model robustness. Furthermore, the *F*1 Score is used as a balanced metric that combines Precision and Recall, defined as their harmonic mean (Equation (13)). It is particularly useful when seeking a balance between false positives and false negatives.

(13) $$F1=2\cdot \frac{P\cdot R}{P+R}$$

## 3. Experimental Results

### 3.1. Experimental Environment

All experiments were conducted on a Linux server equipped with an NVIDIA Tesla V100 SXM2 GPU (32 GB VRAM) using driver version 535.154.02, CUDA 11.8, and cuDNN 8.9. The code was implemented in Python 3.8.2 with PyTorch 2.4.1 and the Ultralytics framework. To ensure reproducibility, a fixed random seed was set. The input images were resized to 640 × 640 pixels. We initialized the model with pre-trained YOLOv11n weights and trained it for 500 epochs with an early stopping patience of 10. The training used a batch size of 16 and the AdamW optimizer with an initial learning rate of 0.01, momentum of 0.937, and weight decay of 0.0005. All other hyperparameters followed the default configurations of YOLOv11. The training environment is mainly summarized in Table 3. The codes of the original YOLOv11n is directly obtained from open source (website [30]).

### 3.2. Enhanced Convergence Robustness of MSFN-YOLOv11

To intuitively evaluate the training effectiveness of the models under different noise conditions, this study conducted a visual analysis of the training processes of both the original YOLOv11 model and our improved MSFN-YOLOv11 model. The visualization metrics include loss/accuracy curves and F1-confidence curves.

(1) Analysis of the loss/Accuracy Graph

**Figure 8:**
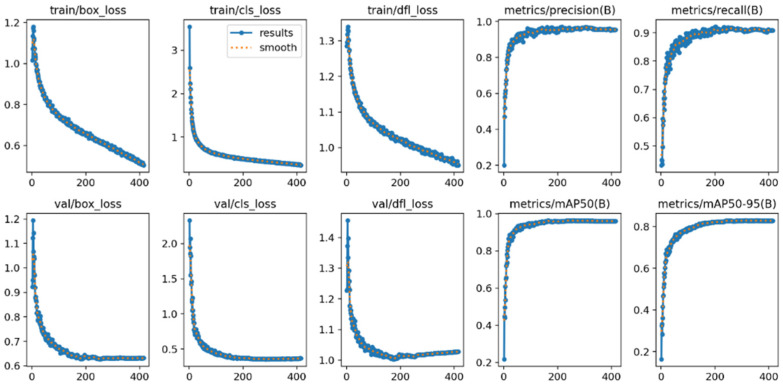
Loss/Accuracy metrics graph of the original YOLOv11.

**Figure 9:**
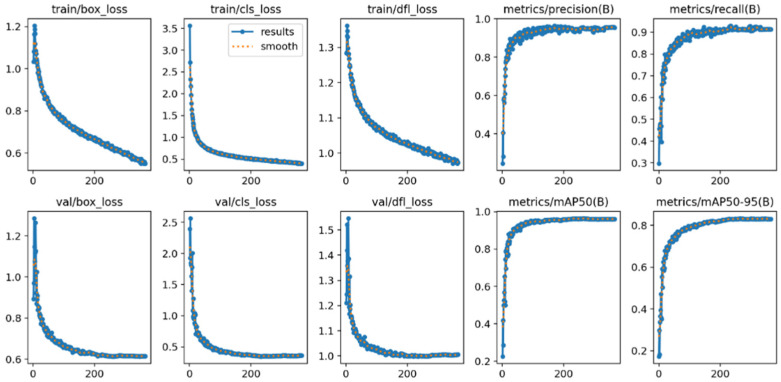
Loss/Accuracy metrics graph of MSFN-YOLOv11.

Figure 8 and Figure 9, respectively, illustrate the training process curves for both the baseline YOLOv11n and the improved MSFN-YOLOv11n model on a noise-containing dataset. These figures depict trends in loss functions, including box loss, classification loss (cls loss), and distribution focal loss (DFL loss), as well as detection accuracy based on Intersection over Union (IoU) thresholds of ≥0.5. Collectively, these metrics reflect the model’s learning behavior and generalization performance under conditions of feature degradation. The training classification loss of the improved model commenced at 3.5, which is slightly higher than the original model’s loss of 3.0. However, it decreased rapidly over the epochs, converging to approximately 0.5, thus matching the performance of the original model. This improvement indicates more efficient feature learning due to the integration of the MSFN module. Regarding validation loss, both models achieved similar final values (val/box_loss ≈ 0.6); nonetheless, the improved model exhibited a smoother curve with reduced fluctuations, signifying enhanced generalization capability and greater robustness to noise and sample variability. Overall, the loss and accuracy curves confirm that the MSFN-YOLOv11 outperforms the original model, achieving both faster convergence and greater stability.

(2) *F*1–Confidence Curve Analysis

**Figure 10:**
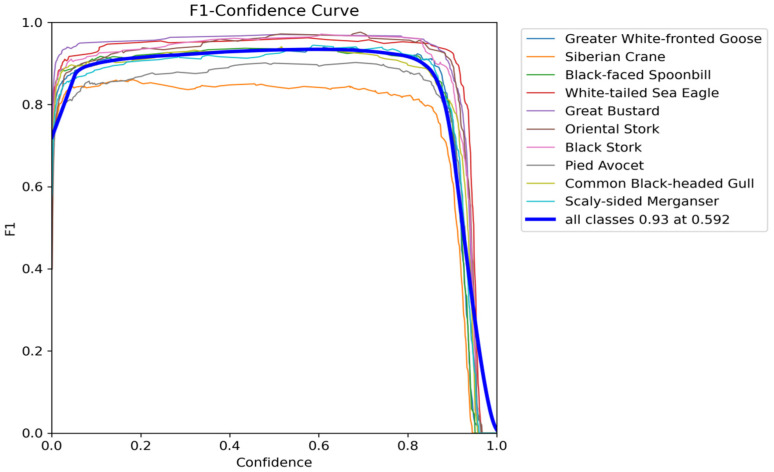
*F*1–Confidence Curves of the original YOLOv11.

**Figure 11:**
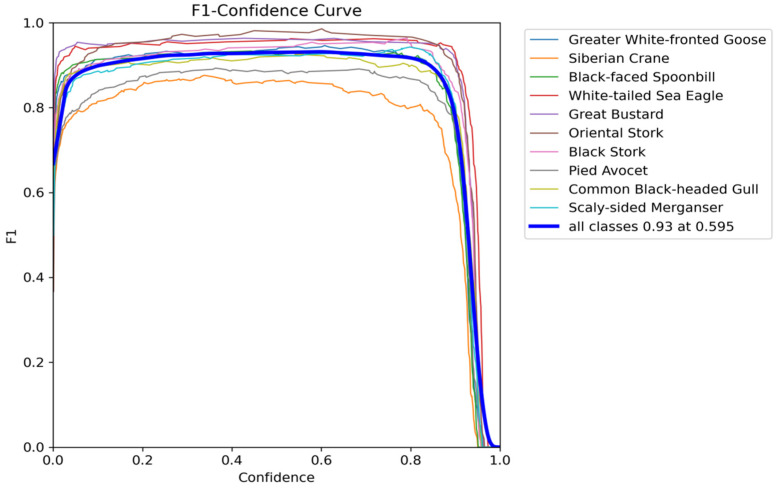
*F*1–Confidence Curves of the MSFN-YOLOv11.

The *F*1–confidence curve provides a two-dimensional evaluation of detection quality through parameterized window scanning. Figure 10 and Figure 11, respectively, illustrate the confidence curves for both the original YOLOv11 and the improved model, respectively. The *F*1 score, defined as the harmonic mean of precision and recall, serves as a metric for assessing the overall performance of the model across various confidence thresholds. Both models demonstrated high *F*1 scores at elevated confidence levels, indicating an effective balance between precision and recall in object detection. Notably, when the *F*1 score reached 0.93, the confidence threshold of the improved MSFN-YOLOv11 model increased from 0.592 to 0.595, reflecting enhanced stability in high-confidence predictions and a decrease in low-confidence misjudgments. Within the 0.6~0.8 confidence interval, the fluctuations of the curve were minimized, thereby reducing misjudgments attributed to feature blur, which renders it more suitable for species classification tasks in ecological monitoring.

### 3.3. Overall Performance Analysis of MSFN-YOLOv11 for Bird Detection

(1) Superior Performance and Efficiency of MSFN-YOLOv11

To demonstrate model’s overall performances for various models, four commonly used evaluation metrics in this study were used to access the identification accuracy: Precision (*P*), Recall (*R*), Mean Average Precision (*mAP*) and Training time. The comparative results are demonstrated in Table 4. The proposed MSFN-YOLOv11 model demonstrates superior overall performance and notable training efficiency over the baseline YOLOv8 and YOLOv11 models. It achieves the highest scores across key metrics: a precision of 96.3% (surpassing YOLOv8 by 3.9% and YOLOv11 by 1.3%), a recall of 97.0%, and a *mAP*@50 of 96.4%. Furthermore, its *mAP*@50-95 of 83.2% confirms enhanced robustness under more stringent localization criteria. Most notably, MSFN-YOLOv11 required only 2.367 h for training, which is 18.8% and 18.0% faster than YOLOv8n (2.914 h) and YOLOv11n (2.886 h), respectively, underscoring its significant computational advantage.

The methodological evolution from YOLOv8 to our model showcases a clear path of architectural improvement. YOLOv8 provides a solid baseline, which YOLOv11 enhances with superior feature extraction via C3K2 and C2PSA modules [31]. Our MSFN-YOLOv11 represents a critical advancement by integrating the novel Multi-Scale Feedforward Network (MSFN) to form a C2PSA_MSFN block. This targeted innovation directly yields the observed gains in precision, recall, and *mAP*, alongside reduced training time, confirming the MSFN module’s role in boosting generalization and convergence. These attributes make the model exceptionally suited for efficient, real-time bird monitoring in complex wetland environments.

(2) Detection Accuracy of MSFN-YOLOv11 for ten types of birds

**Figure 12:**
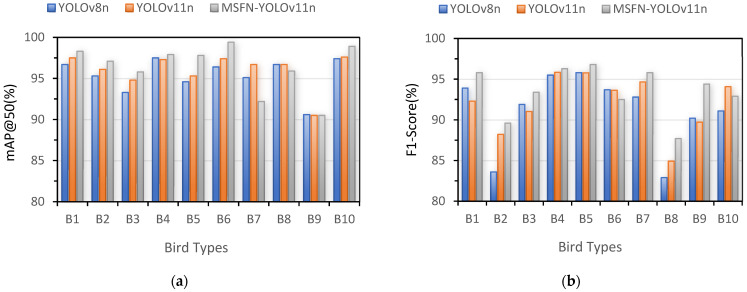
Comparative results of identification accuracy from three algorithms for ten types of birds: (**a**) *mAP*@0.5 (%); (**b**) *F*1-Score (%).

A comparative analysis was further conducted to evaluate the recognition performance disparities across ten distinct bird categories: the Black Stork (B1), Black-faced Spoonbill (B2), Common Black-headed Gull (B3), Great Bustard (B4), Greater White-fronted Goose (B5), Oriental Stork (B6), Pied Avocet (B7), Scaly-sided Merganser (B8), White Crane (B9), and White-tailed Sea Eagle (B10). The quantitative results, detailed in Table 5 and visualized in Figure 12, demonstrate that the proposed MSFN-YOLOv11 model achieves superior or highly competitive detection accuracy compared to the YOLOv8n baseline and the original YOLOv11n. Specifically, MSFN-YOLOv11 attained the highest *mAP*@0.5 in nine out of the ten categories (B1–B6, B8–B10), with a particularly notable performance in category B6, where it reached 99.4%, surpassing YOLOv8n (96.4%) and YOLOv11n (97.4%). In terms of *F*1-Score, which provides a balance between precision and recall, the proposed model also led in seven categories (B1–B5, B7–B9). A significant improvement is observed in B9, where the *F*1-Score rose to 94.4%, a clear gain over the 90.2% and 89.7% values achieved by the other two models, respectively. While MSFN-YOLOv11’s F1-Score was slightly lower in B6 and B10, its performance in the *mAP*@0.5 metric for these categories remained the highest, indicating robust detection capability. Overall, the consistent outperformance of MSFN-YOLOv11 across the majority of bird types underscores its enhanced feature representation and classification stability for avian detection tasks.

### 3.4. Generalization Performance in Validation Experiment

(1) Detection Results of MSFN-YOLOv11 Under Noisy Conditions

**Figure 13:**
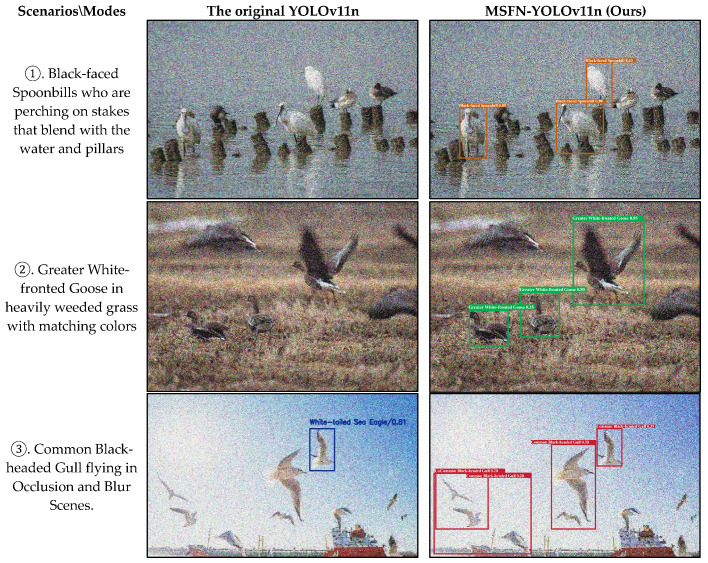
Comparison of Results between MSFN-YOLOv11 and the original YOLOv11.

To scientifically evaluate the detection performance of the improved model in complex scenes, a comparative experiment was conducted using a bird image dataset characterized by typical interference features. The test samples covered three challenging scenarios: (i) birds perching on stakes that blend with the water and pillars (e.g., Figure 13①); (ii) birds in heavily weeded grass with matching colors (e.g., Figure 13②); and (iii) motion-blurred images of birds in flight (e.g., Figure 13③). All images contained noises. The detection results are illustrated in Figure 13. The experimental results indicate that the MSFN-YOLOv11 achieved higher target recognition accuracy under similar background conditions, exhibiting significantly enhanced feature capture capability. In clustered scenes, the original model was susceptible to unrecoverable recognition errors, whereas the improved model yielded more accurate recognition results.

(2) Birds Identification and Application on Real Surveillance Videos

To rigorously evaluate the generalization capability and practical efficacy of the MSFN-YOLOv11 model, we deployed it on multiple field-captured videos featuring four distinct bird species on real surveillance videos from social media (https://www.bilibili.com, accessed on 9 September 2025). As quantitatively summarized in Table 6, the model demonstrated consistent and robust real-time performance across all test scenarios. It achieved an average inference speed of 72 FPS and a mean accuracy of 63.1%, with performance variation (52.6% to 70.5%) directly attributable to specific video challenges such as small target size and complex motion patterns. Notably, the high average confidence level of 69.85% further corroborates the model’s reliable feature extraction and classification ability in diverse, unconstrained wetland environments. These results collectively affirm the strong generalization power of MSFN-YOLOv11 for practical avian monitoring applications.

**Figure 14:**
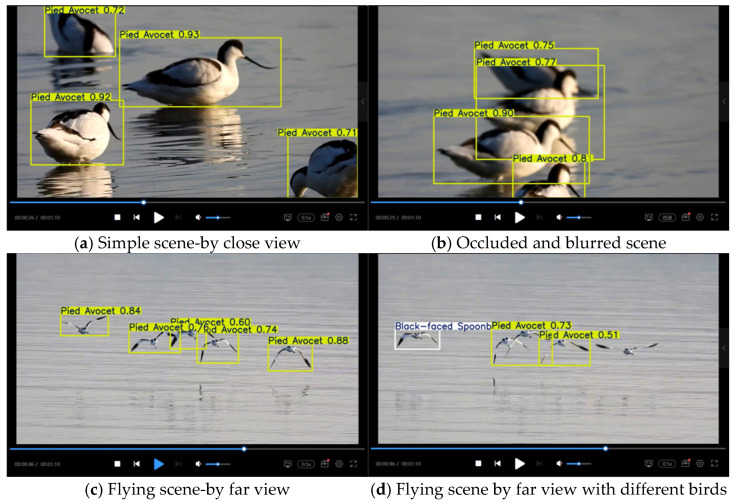
Recognition results based on MSFN-YOLOv11 from filed-captured video.

Figure 14a–d presents visualization recognition results of the MSFN-YOLOv11 model on a real-world surveillance video for Pied Avocets, capturing clustered behaviors such as foraging, perching, and flight under challenging imaging conditions including water surface reflections, motion blur, and occlusion. The model achieves high confidence levels (often exceeding 70%) in open scenes with stationary or slow-moving birds and maintains robust detection (≈60% confidence) under mild blur or reflection. However, in high-speed flight scenarios where birds appear as small, blurred targets with obscured discriminative features, recognition performance declines, leading to occasional false positives and missed detections. These results validate the model’s practical utility in realistic wetland settings, while highlighting a direction for future improvement in handling highly dynamic small targets. Such enhancements could further support ecological applications like tracking migration paths and generating dynamic species distribution maps.

## 4. Discussion

The experimental results demonstrate that the proposed MSFN-YOLOv11 model successfully addresses key challenges in wetland bird recognition, as outlined in our initial contributions. The core of this improvement lies in the novel MSFN module, which enhances multi-scale contextual feature representation through its parallel dilated convolutional paths. This design allows the model to simultaneously capture fine-grained details for small, occluded birds and broad spatial contexts for distinguishing them from cluttered backgrounds like reed beds. Coupled with the channel attention mechanism, which dynamically prioritizes discriminative features (e.g., unique plumage or beak morphology), the model achieves a significant boost in both precision (96.3%) and recall (97.0%). Furthermore, this architectural refinement, by enabling more efficient feature fusion and faster convergence, directly contributed to the 18% reduction in training time. The model requires fewer epochs to learn robust features, as the MSFN provides a stronger multi-scale feature foundation from the earlier stages of training.

Our work also validates the synergistic effect of algorithmic innovation and data-centric strategies. The targeted mixed-noise augmentation, simulating composite disturbances such as motion blur and water surface glare, was crucial for bridging the gap between clean laboratory data and complex field conditions. This strategy, applied to our high-quality, region-specific dataset, directly enhanced the model’s robustness, as evidenced by its high *mAP*@50-95 of 83.2% on the noisy test set and reliable performance in real-world videos. When compared to prior studies, which often focused on static images or specific noise types, our model shows superior generalization in dynamic video streams under composite disturbances. This confirms that our approach effectively fulfills the contribution of providing a practical technical solution for real-time monitoring.

Despite the strong performance, limitations remain, pointing to valuable future directions. The model’s capability on extremely small, fast-flying birds in dense flocks can be further improved. Future work will focus on integrating temporal information from video sequences to leverage motion cues for tracking these challenging targets. Additionally, expanding the dataset to include more species and a wider variety of extreme conditions (e.g., night, heavy rain) will enhance generalizability across different wetland ecosystems. Ultimately, the MSFN-YOLOv11 model establishes a robust foundation for future developments in automated ecological monitoring, with the potential for deployment on edge devices to enable large-scale, real-time biodiversity conservation.

## 5. Conclusions

This study proposes the MSFN-YOLOv11 model to address the challenges of real-time bird detection in complex wetland environments. The core innovation lies in the novel Multi-Scale Feedforward Network (MSFN) module, which enhances multi-scale contextual information capture and discriminative local feature focus through parallel dilated convolutions and channel attention mechanisms. Experimental results on a Dongting Lake bird dataset with synthetic noise demonstrate that the model achieves 96.4% mAP@50 and 97.0% recall while improving training efficiency by 18%, attributed to the MSFN’s efficient feature fusion and faster convergence. The model’s compact architecture and demonstrated robustness provide a solid foundation for strong quantization and deployment on resource-constrained edge devices.

The main contributions include an advanced feature fusion design for lightweight detectors and targeted data enhancement strategies, offering a practical solution for automated avian monitoring. Future work will focus on developing multi-modal systems that integrate optical, acoustic, and remote sensing data; exploring temporal modeling techniques for the continuous analysis of avian behavior; and optimizing model edge deployment capabilities for drones and other terminals. These efforts will ultimately establish a foundation for an integrated “air-space-ground” ecological sensing network, providing technical support for global biodiversity conservation.

## Figures and Tables

**Table 1:** Number of Bird Samples.

Bird Types	Bird Name	Images	Samples	
			Training Set	Test Set
B1	Black Stork	489	572	142
B2	Black-faced Spoonbill	514	488	154
B3	Common Black-headed Gull	311	523	153
B4	Great Bustard	527	563	140
B5	Greater White-fronted Goose	446	607	150
B6	Oriental Stork	413	493	107
B7	Pied Avocet	426	515	136
B8	Scaly-sided Merganser	445	511	147
B9	White Crane	342	599	171
B10	White-tailed Sea Eagle	532	531	122
/	Total	4540	5402	1422

**Table 2:** Quantity of Noisy Pictures.

Noise Types	Standard Deviation (σ)	Ratio	Quantity/Sheet
Mild Gaussian Noise	0.2	0.10	427
Medium Gaussian Noise	0.3	0.12	512
Severe Gaussian Noise	0.4	0.03	126

**Table 3:** Experiment Environment Configuration.

Environment	Configuration Name	Configuration Parameter
Hardware environment	CPU	Intel(R)Xeon(R) Gold 5220 CPU @ 2.20 GHz
	temporary memory	62 G
	GPU	NVIDIA_Tesla_V100_SXM2_32_GB
	video memory	32 GB
Software environment	operating system	Linux
	PyTorch	v2.4.1
	CUDA	v11.8
	Python	v3.8.2
	NVIDIA Driver	535.154.02
Training Settings	Optimizer	SGD
	Epochs/Batch/Size	500/16/640 × 640
	Seed	0 (deterministic = True)

**Table 4:** Comparison of training outcomes between YOLOv8 and MSFN-YOLOv11 models.

Models	*P* (%)	*R* (%)	*mAP*@50 (%)	*mAP*@50-95 (%)	Training Time/h
YOLOv8n (Baseline)	92.4	96.0	95.6	81.7	2.914
YOLOv11n (Original)	95.0**_+2.6_**	96.0**_+0.0_**	96.1**_+0.5_**	82.9**_+1.2_**	2.886**_−0.028_**
MSFN-YOLOv11n (Ours)	96.3**_+3.9_**	97.0**_+1.0_**	96.4**_+0.8_**	83.2**_+1.5_**	2.367**_−0.547_**

Notation: ‘+’ and ‘−’ are improvement from other model vs. YOLOv8 baseline.

**Table 5:** Results of detection accuracy with three models under ten types.

Bird Types	*mAP*@50 (%)			*F*1-Score (%)		
	YOLOv8n (Baseline)	YOLOv11n (Original)	MSFN-YOLOv11n (Ours)	YOLOv8n (Baseline)	YOLOv11n (Original)	MSFN-YOLOv11n (Ours)
B1	96.7	97.5	98.3	93.9	92.3	95.8
B2	95.3	96.1	97.1	83.6	88.2	89.6
B3	93.3	94.8	95.8	91.9	91.0	93.4
B4	97.5	97.3	97.9	95.5	95.8	96.3
B5	94.6	95.3	97.8	95.8	95.8	96.8
B6	96.4	97.4	99.4	93.7	93.6	92.5
B7	95.1	96.7	92.2	92.8	94.7	95.8
B8	96.7	96.7	95.9	82.9	84.9	87.7
B9	90.6	90.5	90.5	90.2	89.7	94.4
B10	97.4	97.6	98.9	91.1	94.1	92.9

**Table 6:** Birds Identification results based on MSFN-YOLOv11 in the different videos.

Videos (Birds)	Frame Count	*FPS* (f/s)	Accuracy (%)	Average Confidences (%)	Inference Time (ms)	Tracking or Smoothing
Video1 (Pied Avocet) [32]	1750	70	62.8	71.1	11.43	Smoothing
Video2 (Black Stork) [33]	987	85	66.5	76.8	9.37	Smoothing
Video3 (Oriental Stork) [34]	1800	68	52.6	52.2	11.35	Smoothing
Video4 (Scaly-sided Merganser) [35]	2047	65	70.5	79.3	13.29	Smoothing
Statistics (Average)	1646	72	63.1	69.85	11.36	Smoothing

## Data Availability

The raw data supporting the conclusions of this article will be made available by the authors upon request.
